# Assessment of an exhaled breath test using ultraviolet photoionization time-of-flight mass spectrometry for the monitoring of kidney transplant recipients

**DOI:** 10.1186/s43556-023-00130-6

**Published:** 2023-06-24

**Authors:** Shijian Feng, Chengfang Xiang, Yushi He, Zhuoya Li, Zhongjun Zhao, Bohan Liu, Zhaofa Yin, Qiyu He, Yanting Yang, Zhongli Huang, Tao Lin, Wenwen Li, Yixiang Duan

**Affiliations:** 1 Department of Urology and Institute of Urology, Laboratory of Reconstructive Urology), West China Hospital, Sichuan University, Organ Transplantation Center, Chengdu, People’s Republic of China; 2 College of Chemistry, Sichuan University, Chengdu, 610064 People’s Republic of China; 3 West China School of Public Health and West China Fourth Hospital, Sichuan University, Chengdu, 610041 People’s Republic of China; 4 School of Mechanical Engineering, Sichuan University, Chengdu, 610064 People’s Republic of China

**Keywords:** Kidney transplantation, Breath test, Noninvasive monitoring

## Abstract

Continuous monitoring for immunosuppressive status, infection and complications are a must for kidney transplantation (KTx) recipients. Traditional monitoring including blood sampling and kidney biopsy, which caused tremendous medical cost and trauma. Therefore, a cheaper and less invasive approach was urgently needed. We thought that a breath test has the potential to become a feasible tool for KTx monitoring. A prospective-specimen collection, retrospective-blinded assessment strategy was used in this study. Exhaled breath samples from 175 KTx recipients were collected in West China Hospital and tested by online ultraviolet photoionization time-of-flight mass spectrometry (UVP-TOF–MS). The classification models based on breath test performed well in classifying normal and abnormal values of creatinine, estimated glomerular filtration rate (eGFR), blood urea nitrogen (BUN) and tacrolimus, with AUC values of 0.889, 0.850, 0.849 and 0.889, respectively. Regression analysis also demonstrated the predictive ability of breath test for clinical creatinine, eGFR, BUN, tacrolimus level, as the predicted values obtained from the regression model correlated well with the clinical true values (*p* < 0.05). The findings of this investigation implied that a breath test by using UVP-TOF–MS for KTx recipient monitoring is possible and accurate, which might be useful for future clinical screenings.

**Supplementary Information:**

The online version contains supplementary material available at 10.1186/s43556-023-00130-6.

## Introduction

Kidney transplantation (KTx) is the optimal treatment for end-stage kidney disease (ESKD) [1], offering significantly improved quality of life and reduced mortality risk compared to dialysis [2–4]. However, KTx recipients require ongoing monitoring due to the sophisticated immunosuppressive regimens they are on, which make them susceptible to infection, cancer, and cardiovascular disease (CVD) [5]. Additionally, individuals with underlying ESKD often have numerous comorbidities that require close monitoring [6].

After the first 3 to 6 months post-transplant, KTx patients are typically seen by general nephrologists or internists who may also handle their comorbidities, depending on the availability of resources near the patient [5]. While transplant nephrologists should be involved in the care of KTx patients for the duration of the graft, limited resources often make this difficult to achieve. Many transplant centers are located in large cities, making it inconvenient for a significant portion of KTx recipients to access. Additionally, the evaluation of new ESKD patients who require KTx and the management of post-transplant complications can be time-consuming for transplant nephrologists. Some tests are only available in special transplant centers, which can result in KTx recipients having to travel long distances, leading to unforeseen consequences [7]. Hence, there is an urgent need for a convenient and accurate screening tool that can be used for monitoring KTx recipients.

Conventional monitoring approaches, including blood sampling and kidney biopsy, are both invasive, resources-intensive, and pose risks of infection and bleeding. These risks can be life-threatening and cause significant burden to KTx recipients. As a result, less or non-invasive and more economical approaches have emerged [8]. Currently, there are three commercially available diagnostic kits are available on the market: CareDx's AlloSure and Natera's Prospera identify donor-derived cell-free DNA (dd-cfDNA) in blood, and Transplant Genomics' Trugraf identifies differentially expressed genes [9, 10]. However, each of these tests still requires blood sampling and costs approximately $3000 per test, moreover, their efficacy has yet to be validated [11].

Compared to sampling the interior environment sampling such as blood, the exterior environment may provide a more comprehensive picture of an organism's overall status. At the onset of a disease, the excess water, salt, electrolyte, proteins, DNAs, and RNAs are excreted into the exterior environment to maintain homeostatic balance [12]. In this circumstance, sampling the interior environment might miss early signals or markers [13]. Thus, the exterior environment, which allows for early detection, has advantages for advantages for identifying biomarkers to monitor KTx recipients. Exhaled breath and urine are the most available resources for noninvasive biomarkers. Exhaled breath, in comparison to urine, is more convenient and easier to obtain.

Human exhaled breath is composed mainly of nitrogen, oxygen, carbon dioxide, water vapor, volatile organic compounds (VOCs), and other inert gases. Among these, VOCs are considered a representative of body metabolic status because most of them are synthesized by the human body [14]. In the 1970s, Pauling et al. used gas chromatography technology to examine VOCs from exhaled human breath [14]. They found that approximately 200 VOCs existed within exhaled human breath [14]. Since then, VOC changes have been tested as biomarkers for several different diseases. For example, Rowland et al. tested 10 type I diabetic mellitus patients who exhaled breath and identified 100 significant VOCs. They also claimed that the VOC methyl nitrate could be used as a predictor of blood glucose [15]. Other reports also claimed that VOCs can be used to diagnose lung cancer and breast cancer [16, 17]. Although VOC examination has many advantages, major issues still need to be addressed. First, there is a lack of unified and standardized analysis methods cross different facilities, which can lead to different results for the same issue. Second, there is a lack of simple and efficient techniques for the effective separation and identification of VOCs in exhaled breath samples. Compared with chemical tests that are already widely used in blood and urine analysis, the current basic instruments for exhaled breath analysis (e.g., mass spectrometry) are more complex and require specialized operators. Third, the physiological significance and metabolic pathways of many VOCs in exhaled breath are still unclear, and one VOC may respond to several disease states. This metabolic uncertainty hinders the application of exhaled breath analysis to real clinical practice.

Overall, KTx recipients need constant kidney function monitoring and traditional methods are far from meeting the current need. Therefore, despite these above limitations, we hypothesized that a machine capable of non-invasively, easily and conveniently collecting and analyzing VOCs could significantly contribute to the monitoring of post-transplantation KTx recipients. In our current study, we used real-time online ultraviolet photoionization time-of-flight mass spectrometry (UVP-TOF–MS) to analyze VOCs in exhaled breath, which proved to be a feasible and noninvasive monitoring device for KTx recipients.

## Results

### Study overview and baseline characteristics

Of all the 175 KTx participants, 120 (69%) were male, with a mean (SD) age of 34.4 (± 10.6) years. All recipients were confirmed as KTx recipients by validation with Chinese Transplantation Registrations, and all samples were randomly allocated to the discovery (70%) and validation (30%) data sets. Table 1 showed the baseline characteristics of all eligible participants. The majority of KTx recipients were of Han nationality (84%). The overall body weight index was 20.9 (± 3.0). The median dialysis duration before KTx was 30.8 (± 32.2) months. The KTx recipients also included ABO-incompatible KTx and sensitized recipients, and all causes leading to end-stage renal disease were also included. The majority of KTx recipients received triple immunosuppressive therapy, including tacrolimus, mycophenolate mofetil, and steroids. In general, the KTx recipients who participated in the current study may represent the general KTx recipient population.

**Table 1:** basic characteristics of kidney transplant recipients

Category	N/Mean	%/SD
Male/ Female n (%)	120/55	69%/31%
Race		
Han n (%)	147	84.0%
Tibetan n (%)	11	6.3%
Tu n (%)	9	5.1%
Yi n (%)	7	4.0%
Others n (%)	1	0.6%
Age (year)	34.4	10.6
Weight (Kg)	58.6	9.3
Height (m)	1.7	0.1
BMI (Kg/m2)	20.9	3.0
Hypertension n (%)	126	72.0%
Systolic blood pressure	138.2	15.8
Diastolic blood pressure	92.6	13.4
Mean arterial pressure	107.8	12.9
Pulse pressure Mean	45.7	13.3
ESRD duration (month)	42.8	41.9
Dialysis duration (month)	30.8	32.2
Smoking history n (%)	11	6.3%
Drinking history n (%)	12	6.9%
HLA class I antibody screening negative n (%)	110	62.9%
HLA class II antibody screening negative n (%)	128	73.1%
Secondary transplant n (%)	17	9.7%
Primary nephropathy		
IgA nephropathy n (%)	17	9.7%
Hypertensive nephropathy n (%)	11	6.3%
Diabetic nephropathy n (%)	9	5.1%
Unknown n (%)	137	78.3%
Other diseases		
Renal hypertension n (%)	116	66.3%
Renal anemia n (%)	37	21.1%
Renal cyst n (%)	23	13.1%
Renal osteodystrophy n (%)	12	6.9%
Coronary atherosclerosis n (%)	4	2.3%
Immunosuppressant		
Tacrolimus n (%)	175	100%
Mycophenolate n (%)	123	70.3%
Steroid n (%)	54	30.1%

### UVP-TOF–MS exhibited satisfying efficacy in exhaled breath tests

**Fig. 1:**
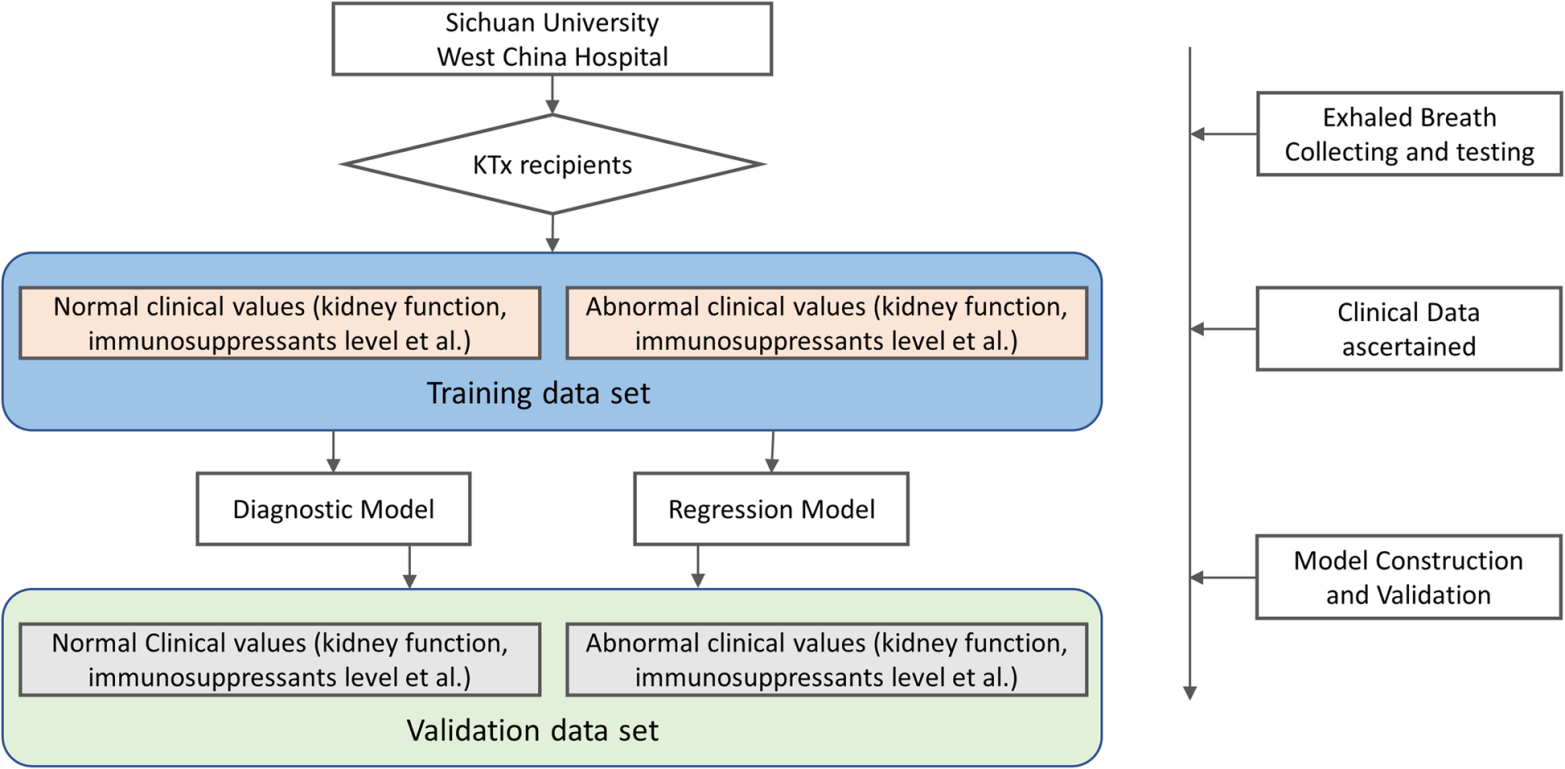
Flow diagram of study design

All people who we approached to join in the study agreed to participate the test, resulting in a 100% patient acceptance rate, and we were able to successfully collect samples from every single KTx recipient. No negative effects or discomforts were observed while collecting breath samples. Peaks with m/z < 29 and > 300 were excluded from the data analysis due to their low content. The mass spectrum (m/z 29 ~ 300) of breath sample was shown in Fig. 1. Each mass spectra were accumulated for 50 s, and it only took 1 min to detect each sample.

UVP-TOF–MS is a promising tool for identifying malfunctioning kidney transplant recipients.

As a screening tool, the primary objective was to identify abnormal situations from normal ones. Therefore, we developed a series of classification models based on exhaled VOCs. For KTx recipients, kidney function was the primary concern. Serum creatinine (SCr), estimated glomerular filtration rate (eGFR), and blood urea nitrogen (BUN) were the most commonly used clinical parameters for kidney function.

**Fig. 2:**
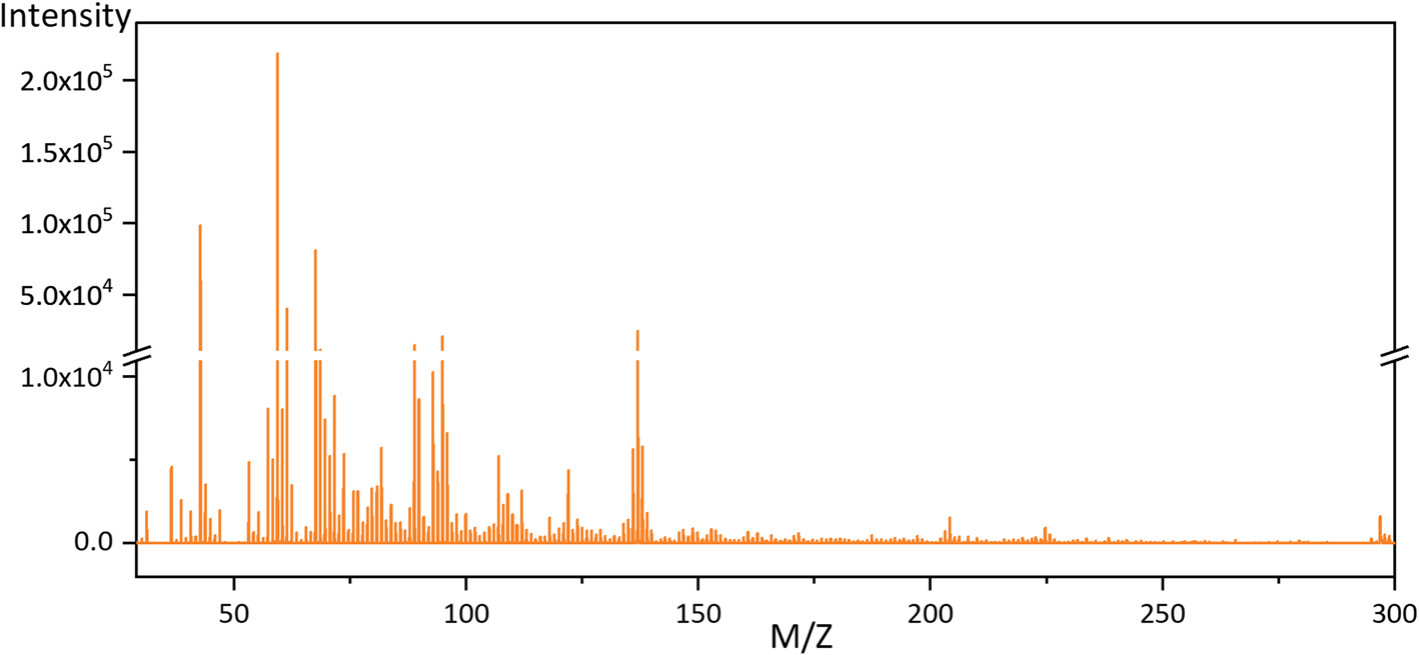
Mass spectrogram of breath sample using UVP-TOF-MS

The SCr threshold was set at 108 µmol/L, and values below 108 µmol/L were considered normal. In the enrolled population of this study, there were 46 subjects with normal SCr values and 129 subjects with abnormal values. By using 200% SMOTE resampling to produce a balanced dataset (138 normal-129 abnormal), we generated a classification model with 70% data and validated it with the rest 30% data. Figure 2a showed that area under the curve (AUC) of the receiver operating characteristic (ROC) curve was 0.889 for SCr classification with validation dataset (normal, *n* = 41; abnormal, *n* = 39), indicating a satisfying classification efficacy (sensitivity = 0.846, specificity = 0.805). Furthermore, a classification model with 46 normal and 46 abnormal SCr subjects (under-sampling for the majority) produced an AUC value of 0.878 (sensitivity = 0.857, specificity = 0.857). These results suggested that the classification model performed well in separating normal SCr values from abnormalities by both oversampling or under-sampling, so exhaled VOCs could be used as a monitoring method for warning of abnormal serum creatinine status.eGFR was set as normal (> 90 ml/min/1.73 m2), mildly decreased (60–89 ml/min/1.73 m2), moderately decreased (30–59 ml/min/1.73 m2), and severely decreased (< 30 ml/min/1.73 m2) [18]. We firstly tested whether exhaled VOCs could differentiate each eGFR category using a multi-classification model. However, the multi-classification model demonstrated poor efficiency (overall accuracy = 0.51). We then tested whether VOCs could differentiate mild impairment from moderate and severe impairments. As shown in Fig. 2b, the ROC (AUC = 0.850) revealed good classification result for classify mild (normal + mild decrease, *n* = 78) and serious status (moderate + severe decrease, *n* = 97) with sensitivity 0.828 and specificity 0.782.

BUN was defined as 50 mmol/L, with 50 normal and 125 abnormal values. The classification model by using 150% SMOTE resampling (125 normal, 125 abnormal) for the minority produced an AUC value of 0.847 (Fig. 2c, sensitivity = 0.811, specificity = 0.703). In addition, we developed a classification model by under-sampling for the majority (50 normal, 50 abnormal) produced an AUC value of 0.836 (sensitivity 0.800, specificity 0.800).

**Fig. 3:**
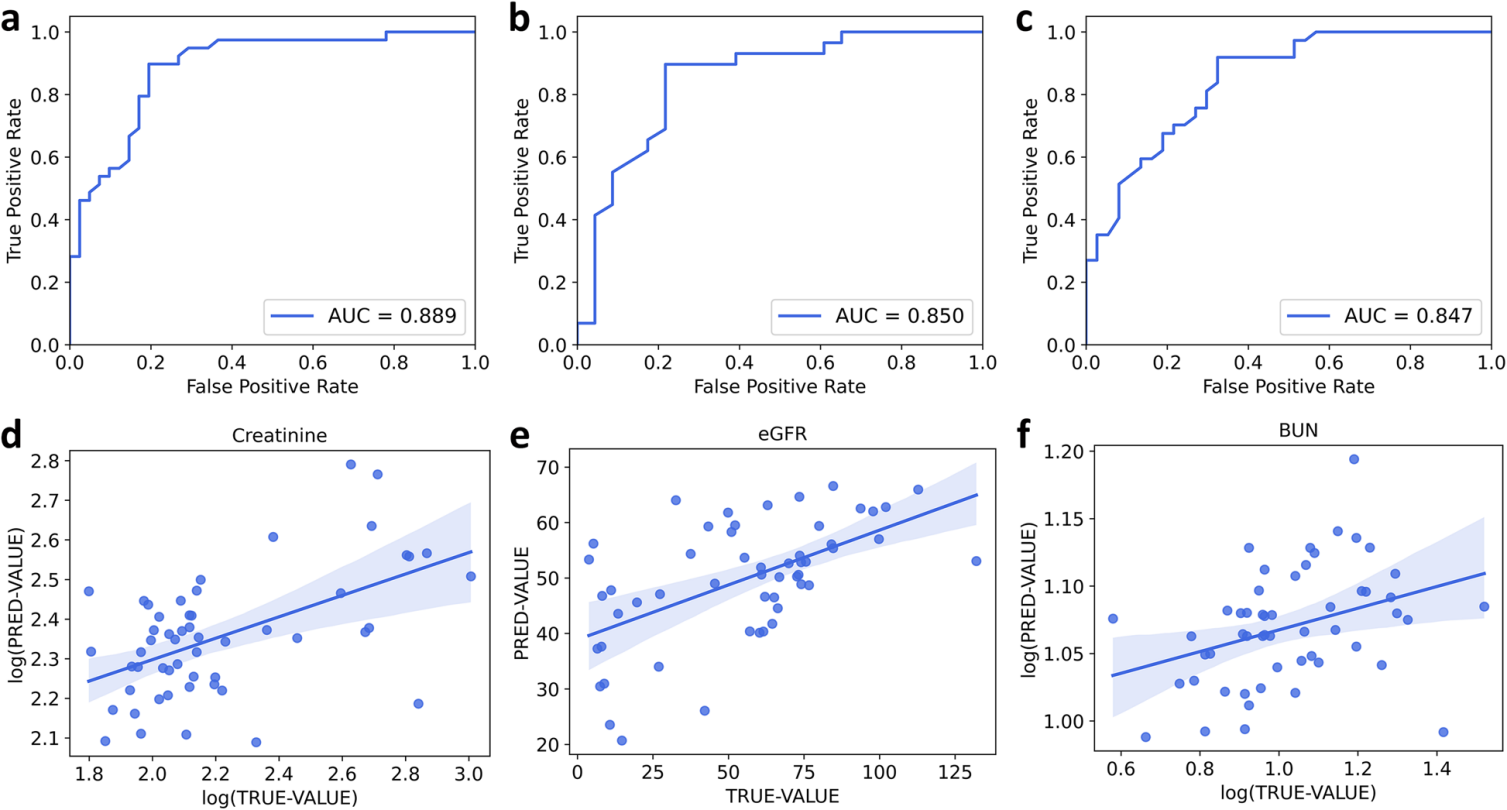
Classification and regression efficacy of essential kidney function parameters based on exhaled VOCs

In addition to kidney function, immunosuppressants is also crucial in monitoring KTx recipients. High levels of tacrolimus could lead to severe kidney toxicity, while low levels could not achieve adequate immune suppression. It has to be closely monitored between 5 ~ 8 ng/ml. We tried a multi-classification model for three categories, which demonstrated a poor accuracy of 0.56. Therefore, to achieve higher efficacy for clinical usage, we then generated four two-classification models based on exhaled VOCs to identify low or high tacrolimus levels from normal levels. As shown in Fig. 3a-d, the validation results demonstrated that VOCs can successfully identify low or high tacrolimus levels from normal levels (low, *n* = 47; normal, *n* = 38; high, *n* = 63). Overall, RF classification models based on exhaled VOCs by using UVP-TOF–MS could successfully identify abnormal KTx recipients, and the classification results were summarized in Table 2.

**Table 2:** Results of classification and regression models for KTx monitoring

Parameters	Classification					Regression^c^
	ROC	Sensitivity	Specificity	Sampling^a^	NO of subjects	
Creatinine	0.899	0.846	0.805	SMOTE	138 normal-108 high^b^	*r*=0.51,
	0.878	0.857	0.857	Random	46 normal-46 high^b^	*p*<0.001
eGFR	0.85	0.828	0.782	-	78 (normal + mild)-97 (moderate + severe)^b^	*r*=0.567, *p*<0.001
BUN	0.847	0.811	0.703	SMOTE	125 normal-125 high^b^	*r*=0.269, *p*=0.051
	0.836	0.8	0.8	Random	50 normal-50 high^b^	
Tacrolimus	0.856	0.923	0.75	-	38normal-63high^b^	*r*=0.581, *p*<0.001
	0.806	0.75	0.778	-	38normal-47low^b^	
	0.852	0.8	0.85	Random	63(normal + low)-63high^b^	
	0.75	0.571	0.714	Random	47low-47(normal + high)^b^	

^a^Sampling method SMOTE is used for over-sampling and random is used for under-sampling of unbalanced dataset when doing classification

^b^These groups act as positive classes in the classification models

^c^Regression results show the correlations between KTx clinical true values and model predicted values based on the validation dataset

The regression models of UVP-TOF–MS in exhaled breath correlated well with clinical parameters.

Encouraged by the excellent classification ability of exhaled VOCs, we then built regression models to determine whether exhaled VOCs could predict the actual clinical parameters. All the regression models were trained by 70% of the dataset and validated by 30% of the dataset. For kidney function parameters, the regression models exhibited acceptable correlations between clinical true values and model predicted values for creatinine (*r* = 0.511, *p* < 0.001), eGFR (*r* = 0.567, *p* < 0.001), and BUN (*r* = 0.269, *p* = 0.051). Tacrolimus levels also demonstrated good correlation efficacy (*r* = 0.518, *p* < 0.001). The correlations between true values and model predicted values were shown in Fig. 2d-f and Fig. 3e, respectively.

### Acetone was identified as significant exhaled content for KTx Monitoring

**Fig. 4:**
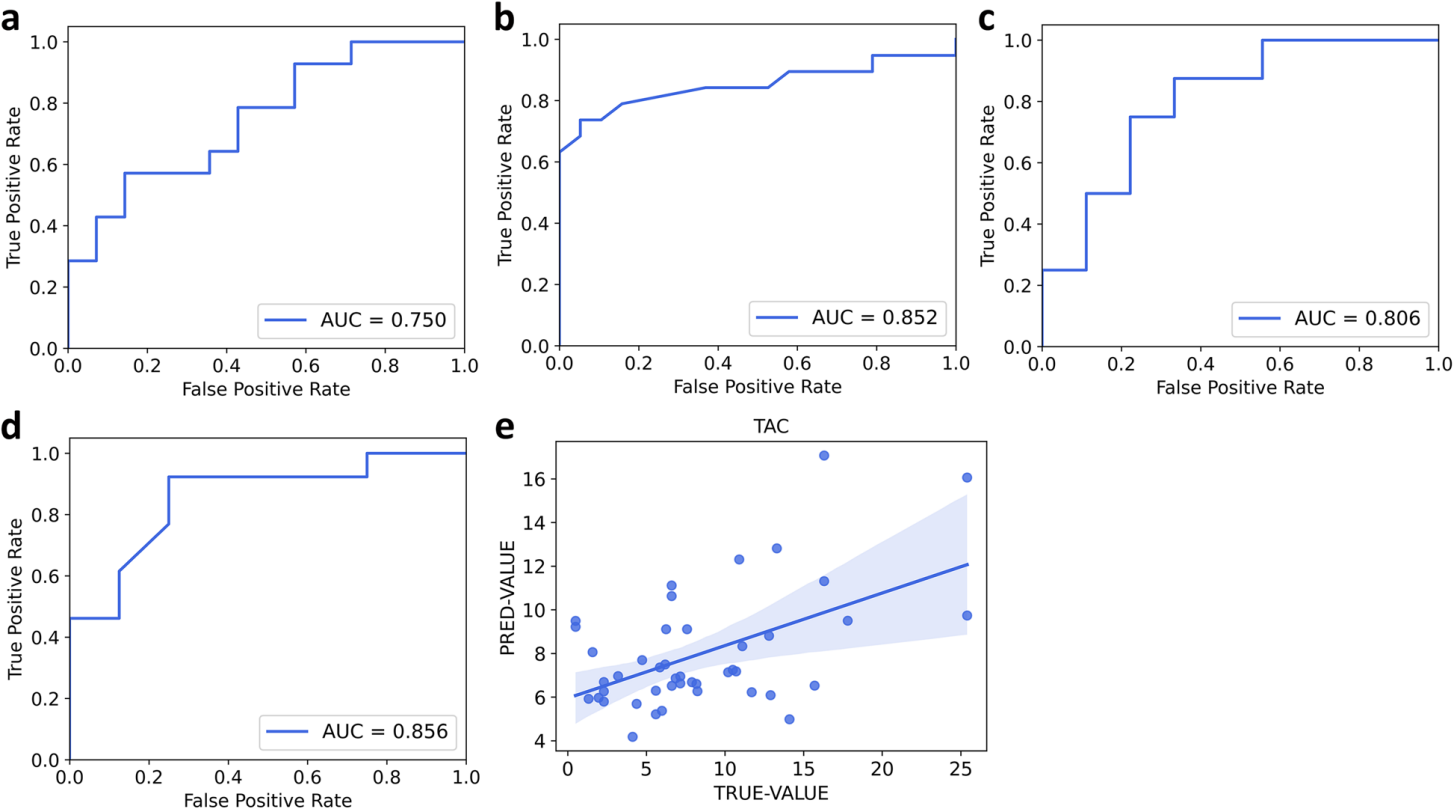
Classification and regression efficacy of immunosuppressants (tacrolimus) concentration based on exhaled VOCs

The above investigations have demonstrated that by using UVP-TOF–MS for the examination of VOCs from exhaled breath could either identify abnormal KTx recipients or correlate with clinical parameters. Further investigation of which VOCs contributed to the classification of KTx recipients would shed some light on the potential mechanism underlying the phenotype. A total of 52 features were found significant difference between high and low SCr gourps. The top 5 features (*p* < 0.01) included m/z 44, m/z 61, m/z 76, m/z 147, m/z 237 (Fig. 4a). The different exhaled breath VOCs between normal/mild decreased and moderate/severe decreased eGFR were also identified (68 features); the top 5 features (*p* < 0.001) were m/z 43, m/z 61, m/z 76, m/z 142, m/z 237 (Fig. 4b). In addition, 5 top VOC features, including m/z 88, m/z 198, m/z 200, m/z 261, m/z 275 (Fig. 4c), were found to be significant based on BUN levels. For tacrolimus levels, 44 VOC features showed significant differences among low, normal and high groups, and the top 5 VOCs (*p* < 0.01) were m/z 39, m/z 41, m/z 69, m/z 83, m/z 142 (Fig. 4d). The possible compounds corresponding to each m/z were shown in Table 3. Amone these different metabolic contents, acetone was found to be the crutial elements. Bioinformatic investigation based on the metabolic contents revealed above, we also identified that the butanoate metabolism, and propanoate metabolism were significantly affected metabolic pathways (Supplementary Fig. 5–7). This also confirmed the importance of acetone as a biomarker in monitoring kidney function.

**Table 3:** exhaled compounds corresponding to each m/z (^#^compounds were identified by standards, the mass spectra of standards were given in supplementary file)

m/z	Chemicals (CAS)	MW	Ionization mode
39	#n-Propanol (71–23-8)	60.10	M-21
41	#n-Propanol (71–23-8)	60.10	M-19
43	#Acetone (67–64-1)	58.08	M-15
44	Propane (74–98-6)	44.09	M
61	#Vinyl acetate (108–05-4)	86.09	M-25
69	#Vinyl acetate (108–05-4)	86.09	M-17
76	#Carbon Disulfide (75–15-0)	76.14	M
83	#1-Octene (111–66-0)	112.21	M-29
88	#Allyl methyl sulfide (10,152–76-8)	88.17	M
142	#1-Nonanal (124–19-6)	142.24	M
147	#N, N-Dimethylformamide	73.09	2 M + 1
198	C14H30	198.39	M

The results of UVP-TOF–MS also showed positive correlation with other clinical parameters.

Liver function and other clinical parameters were also measured. And a regression model was generated and validated as well. Since the data were highly biased based on the discriminative analysis, the classification analysis was not performed. The regression data demonstrated that clinical true values of direct bilirubin, alanine aminotransferase, triglycerides and blood glucose were significantly correlated with model predicted values (*r* = 0.349, *p* = 0.01; *r* = 0.344, *p* = 0.011; *r* = 0.338, *p* = 0.013; *r* = 0.323, *p* = 0.018; respectively). In contrast, regression models showed ordinary performance in other clinical parameters, including total bilirubin, indirect bilirubin, total bile acids, aspartate aminotransferase, alkaline phosphatase, glutamyl transpeptidase and uric acid (Supplementary Fig. 1). Furthermore, classification model based on exhaled breath test demonstrated good discriminative efficacy for glucose status distinction (Supplementary Fig. 2). Seven exhaled VOCs showed significant difference between normal and abnormal blood glucose status (Supplementary Fig. 3).

## Discussion

Exhaled breath analysis has garnered significant attention in recent years, and it might become the preferred method of choice for various applications in medical diagnostics and customized treatment monitoring in the future [19]. The UVP-TOF–MS for the VOCs analysis sheds light on a person's metabolic status through online analysis of exhaled breath is even more significant and advantageous because the results are available immediately rather than having to send samples to a lab and wait for test time [20]. Breath may be obtained in literally infinite volumes, its analysis is painless for the patient being monitored, and its online analysis enables the continuous monitoring of metabolic health, disease development, and medicine concentration in short time intervals. Furthermore, in certain critical situations, such as the emergency room, for proper treatment of a dangerous infection such as pneumonia, when consumption of potentially dangerous drugs must be determined, in doping control, or in other situations where immediate action is needed, this approach may become indispensable [21]. Exhaled breath analysis could potentially reveal novel biomarkers for diseases, allow disease phenotyping, become a low-cost alternative to established new test approaches, allow bedside or even home monitoring, and allow cost-effective and frequent checks of disease progression, therapy effectiveness, and medication adherence.

However, there are some obstacles to overcome. Exhaled breath analysis is still far from being well-established and standardized. And, currently, it is only commonly utilized in clinical practice for a few applications (lung and liver diseases, diabetes, etc.). With relatively low numbers of participants, many studies might be overfitted, therefore, biomarker candidates discovered in case‒control studies must be validated with adequate sample sizes [22]. Data treatment, particularly data processing and chemical identification, suffers from a lack of standards and reporting. There are a variety of analytical procedures that might be used, however, they all have problems with reproducibility, batch effects, temporal drift, and confounding variables. The requirements for very high sensitivity, large dynamic range to detect metabolites with widely varying concentrations, and chemical selectivity to analyze the complex mixture of compounds found in exhaled breath are at odds with the requirements for very low cost and portability of instrumentation.

The main mass spectrometry methods for detecting VOCs and subsequent analysis were selected ion flow tube mass spectrometry (SIFT-MS), proton transfer reaction mass spectrometry (PTR-MS), and secondary electrospray ionization mass spectrometry (SESI-MS) [23]. This high-resolution mass spectrometry would significantly increase the detection rate of thousands of VOCs and possibly reveal unknown compounds. However, the complex specimen and analysis processing process raised the threshold for clinical screening applications. SIFT-MS has major drawbacks due to its low MS resolution. The concept of PTR-MS itself determines the detectability limit. Proton transfer can only ionize VOCs with a greater proton affinity (PA) than H_2_O, allowing them to be identified using this approach. The fundamental disadvantage of SESI-MS is that it does not yet allow for absolute measurement in the gas phase. In terms of direct mass spectrometry, UVP-TOF–MS has a simpler structure than SIFT-MS, which is easier to maintain and use. And, it has simpler ionization results than PTR-MS, facilitating the understanding of the biological significance of characteristic features. Therefore, UVP-TOF–MS requires no sample preparation or VOC enrichment, and it only takes 60 s to analyze a sample compared to other MS. It also improves resolution, allowing for more accurate VOC detection and quantification. However, a potential disadvantage of UVP-TOF–MS is the aging of the UV lamp, which can cause a decrease in detection sensitivity over time. Due to its high ionization efficiency, high molecular ion yield, and low degree of fragmentation, it is one of the most potent and popular soft ionization methods for online monitoring of trace VOCs.

Therefore, in the current study, we aimed to address the limitation of traditional approaches by UVP-TOF–MS to achieve better performance and a rapid analysis process. To the best of our knowledge, this is the first study evaluating exhaled breath VOCs by using UVP-TOF–MS in monitoring KTx recipients. The results demonstrated promising outcomes and had great potential in clinical transition possibilities. Although the regression based on particular blood analysis was not as perfect as expected, the classification results were more than qualified to be a screening tool.

Although using biomarkers from the exterior environment have many advantages, there are also some major drawbacks that need to be considered. Unlike the interior environment, the exterior environment contains many different types of materials, resulting in a high degree of content diversity and variation in exhaled breath and urine. This diversity and variation can make it challenging to identify the targeted signals. Food, the environment, and other disease-irrelevant elements have some impact on exhaled breath VOCs, which increases the difficulties in classification and regression. To minimize these interferences, we collected exhaled breath before breakfast to exclude food interference. However, on the other hand, this process might hinder wider application of UVP-TOF–MS on screening KTx recipients. Therefore, there is still room for improvement. It is worth noting that certain patients experience inconvenience when giving exhaled breath samples before breakfast. Thus, future research should focus on removing confounding factors in exhaled breath samples. This will not only simplify the whole process but also provide accurate results to both clinicians and patients.

Our study identifies acetone in exhaled breath as a potential biomarker for monitoring kidney transplant (KTx) recipients. Acetone is a type of ketone that can be associated with various metabolic conditions, such as ketosis, lung cancer, dietary fat loss, and diabetes, as demonstrated by previous research. [24–26]. During periods of fasting, exercise, or diabetes mellitus (DM), acetone is one of the three ketone bodies synthesized via metabolic pathways in the liver by breaking down and oxidizing fatty acids to produce energy [27]. Following metabolism, acetone is circulated through the bloodstream and is expelled from the body through exhalation due to its high volatility. Research has indicated that breath acetone is a potential biomarker for diabetes and correlates with blood glucose levels [28]. Additionally, in cachexia, particularly in the end-stage of advanced diseases such as cancer, ketone bodies, including acetone, increase due to protein metabolism [29]. The kidney is a metabolically active organ that primarily derives its energy from fatty acids, lactate, glutamine, and to a lesser extent, glucose. However, during periods of energy stress or certain conditions, ketones play a critical role in maintaining cellular energy balance [30]. Upon filtration by the renal glomeruli, ketones are actively reabsorbed by tubular epithelial cells through the sodium-coupled monocarboxylate transporter 2 (SMCT2), thus minimizing urinary excretion of these valuable compounds during fasting [31]. Moreover, human positron emission tomography studies suggest that the kidney possesses a significant capacity for ketone utilization, indicating exciting prospects for future research in renal medicine. Although clinical data remain insufficient, several emerging cellular and animal experiments proposed that exogenous ketone supplementation might help safeguard the kidney against acute stress, disease conditions, and pathological alterations that occur during aging [32]. In our proposal, we suggest that impaired acetone metabolism by the kidney following transplantation may elevate the concentration of acetone in the bloodstream, resulting in an increase in exhaled breath acetone. While clinical data remain insufficient, human positron emission tomography studies suggest that the kidney possesses significant capacity for ketone utilization, indicating exciting prospects for future research in renal medicine.

Exhaled breath analysis is emerging since it can be easily obtained, the analysis is noninvasive, and the patient being analyzed is not burdened. In brief time intervals, online breath analysis also enables rapid and precise results, which is critical in instances where immediate action is needed. This approach is likely to create a new window into body metabolism and give further diagnostic insight due to the large spectrum of identified metabolites and the sensitivity and selectivity to differentiate particular molecules in exhaled breath. Exhaled breath analysis, we believe, will become the method of choice for diagnosis in a small number of specialized applications in the near future. In the long run, if validation studies with sufficient sample sizes are successful and major industries and diagnostics sectors adopt breath-based technologies, it is easy to imagine that breath-based diagnostics would replace the established tests based on urine or blood samples due to the ease and convenience of obtaining breath samples and the immediate availability of results.

## Methods

### Participant recruitment and study design

**Fig. 5:**
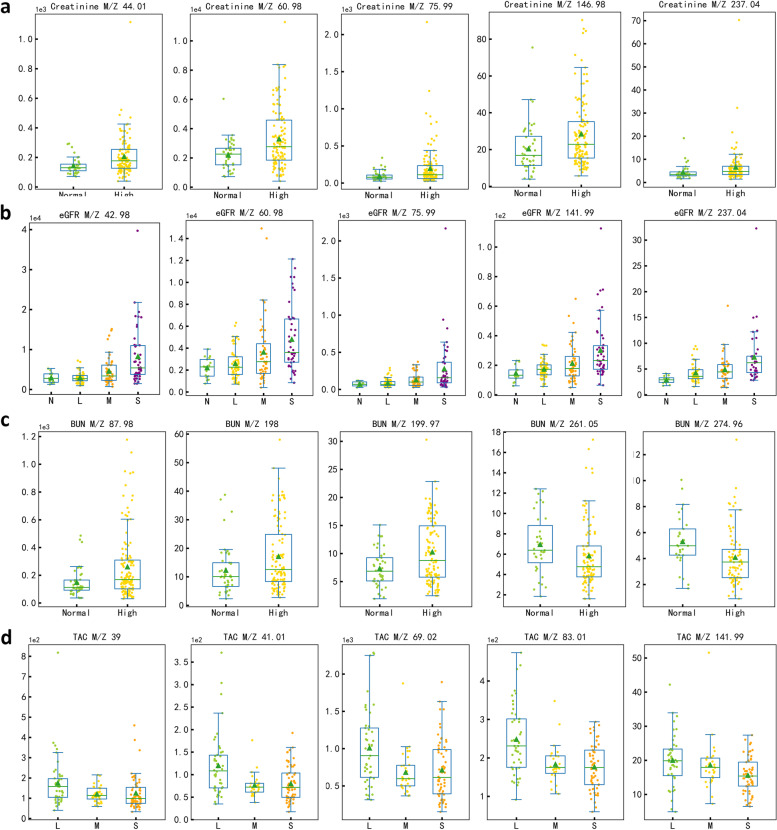
Top 5 significant different exhaled features (m/z) for KTx monitoring. (**A**. Creatinine; **B**. eGFR; **C**. BUN; **D**. Tacrolimus.)

The Standards for Reporting of Diagnostic Accuracy (STARD) reporting standard was followed in this investigation [33]. The whole study design was presented in Fig. 5, and it employed a prospective-specimen collection, retrospective-blinded assessment (PROBE) design [34]. The Ethics Committee Board of West China Hospital, Sichuan University authorized this study, and all subjects provided written informed permission (No.2019–748). KTx recipients were recruited in the outpatient and inpatient Urology Department and Organ Transplant Center of West China Hospital, Sichuan University. In order to include all kinds of different complications post KTx, the only inclusion criterion was set as patients aged over 18 years old. Anyone who did not want to participate in the study was excluded. According to the sample size calculation by G-power, when the given significance level (α) is 0.05, power value (1-β) is 0.90, and effort size (d) is 0.5, the required sample size for each group is 73. And other studies utilizing exhaled breath as a diagnostic test which is similar to our study demonbstrated that sample size over 150 is considered acceptable [35–38]. Therefore, a total of 175 KTx recipients were recruited in our study. Samples were collected from November 1st, 2021 to March 1st 2022.

### Exhaled breath collection

Trained investigators collected all exhaled breath samples according to the collection protocol. All individuals' exhaled breath samples were taken in the morning before breakfast. To collect alveolar air, subjects were required to exhale into a Bio-VOC syringe until they felt mild resistance. The syringe expelled the dead space air from mouth and upper airway and kept the end-tidal breath gas (150 mL), which were then transferred into a 0.5L Tedlar bag. For each patient, three consecutive breaths were collected and injected into the Tedar bag. To exclude environmental contamination, we also collected room air from the same day as a negative control. All VOC tests were performed on the same day as the collection date.

### UVP-TOF–MS

Ultraviolet photoionization time-of-flight mass spectrometry (UVP-TOF–MS, ALIBEN Science & Technology Chengdu) utilized a krypton lamp as the ionization source, which produced photons with an energy of 10.6 eV. Compounds (M) into the ion source will be ionized by photons to produce cations (M +), which is formed as M + hν → M +  + e. Most VOCs have an ionization potential lower than 10.6 eV and can be ionized directly. Mass spectrum peaks with mass-to-charge ratio (m/z) < 500 were detected by UVP-TOF–MS. High efficiency ion analyzer TOF can realize high-throughput and simultaneous analysis of multiple substances. The parameters of UVP-TOF–MS were set as follows: ion-source pressure 95 mbar, ion-source current 1.00 mA, and the signal accumulation time for each sample testing 50 s.

The UVP-TOF–MS is a direct mass spectrometry. Its sample introduction and detection processes are very simple, without preprocessing and parameter optimization. The mass spectrometry is in a vacuum state, with a vacuum degree of 500 Pa. When testing samples, the gaseous components will be automatically absorbed into the ion source and be ionized. Mass spectrometry can achieve a signal acquisition time of 1 s-60 s. The length of the acquisition time is related to the degree of signal accumulation. The longer the acquisition time, the higher the signal. In this work, we used a 50 s acquisition time with about 70 ml to 100 ml gas to perform exhaled breath samples detection.

### Data analysis

The original UVP-TOF–MS data were preprocessed by peak integration and alignment to generate a data matrix of samples (*n* = 175) × features (*n* = 270 from m/z 29 to m/z 300). Then, standardization (Z score) made the data matrix to a common scale without distorting differences in the value ranges or losing information. Feature selection was based on mutual information scores, which measured the contribution of a feature toward reducing uncertainty about the label value. Decision forest regression and decision forest classification were used for regression and classification analysis. Parameter turning for each model was based on a random grid search algorithm. The data matrix was divided into 70% for model training and 30% for model testing. Resampling based on Synthetic Minority Oversampling Technique (SMOTE) and under-sampling by randomly sampling from the majority were used in the classification models to handle the unbalanced dataset. Statistical tests of features between different classes were made through nonparametric tests (Mann–Whitney U test for two class, Kruskal–Wallis test for multiclass), and *p* < 0.05 was considered statistically significant. Data processing was implemented by Python (Edition 2020.1.3) and SPSS (version 21). For bioinformatic investigations, we used KEGGREST software to map the names of metabolites of interest to corresponding KEGG compound numbers. Subsequently, pathway enrichment analysis was conducted using the clusterprofiler software against the background of all KEGG compounds. Finally, the Pathview package was employed to map the metabolites with their respective compound numbers onto the corresponding pathway maps.

## Conclusion

Current breath testing may be a reliable approach for KTx recipient monitoring, and UVP-TOF–MS may provide quick, noninvasive and precise detection of exhaled breath, according to the findings of this diagnostic trial. Exhaled breath has the potential to be used in KTx recipient monitoring.

## Supplementary Information

**Additional file 1: Figure 1.** Correlations between clinical true values and regression model predicted values of blood glucose, triglycerides, uric acid, total bilirubin, direct bilirubin, indirect bilirubin, total bile, aspartate aminotransferase, alanine aminotransferase, alkaline phosphatase, glutamyl transpeptidase of KTx patients. **Figure 2.** RF model for glucose status (135 normal and 40 high) classification by 200% SMOTE resampling (left, 135 normal vs 120 high) and under-sampling (right, 40 normal vs 40 high). **Figure 3.** Significant exhaled compounds (*p*<0.01) between normal (<5.9mmol/L) and high blood glucose status (>5.9 mmol/L). **Figure 4.** Raw mass spectra of standards of significant exhaled compounds. **Figure 5.** KEGG pathway analysis based on different exhaled metabolic contents. **Figure 6.** Crucial different metabolic contents identified by UVP-TOF-MS involved inbutanoate metabolism. **Figure 7.** Crucial different metaboliccontents identified by UVP-TOF-MS involved in propanoate metabolism.

## Data Availability

The data supporting this study are available from the corresponding author upon reasonable request.
