# Supplementary material for: Assessment of an exhaled breath test using ultraviolet photoionization time-of-flight mass spectrometry for the monitoring of kidney transplant recipients
Source: Mol Biomed. 2023 Jun 24;4:19. doi: 10.1186/s43556-023-00130-6 (PMC10289997; doi:10.1186/s43556-023-00130-6)
Supplement: Supplementary file 1 — Additional file 1: Figure 1. Correlations between clinical true values and regression model predicted values of blood glucose, triglycerides, uric acid, total bilirubin, direct bilirubin, indirect bilirubin, total bile, aspartate aminotransferase, alanine aminotransferase, alkaline phosphatase, glutamyl transpeptidase of KTx patients. Figure 2. RF model for glucose status (135 normal and 40 high) classification by 200% SMOTE resampling (left, 135 normal vs 120 high) and under-sampling (right, 40 normal vs 40 high). Figure 3. Significant exhaled compounds (p<0.01) between normal (<5.9mmol/L) and high blood glucose status (>5.9 mmol/L). Figure 4. Raw mass spectra of standards of significant exhaled compounds. Figure 5. KEGG pathway analysis based on different exhaled metabolic contents. Figure 6. Crucial different metabolic contents identified by UVP-TOF-MS involved inbutanoate metabolism. Figure 7. Crucial different metaboliccontents identified by UVP-TOF-MS involved in propanoate metabolism. [file 43556_2023_130_MOESM1_ESM.docx]

**Assessment of an Exhaled Breath Test Using Ultraviolet Photoionization Time-of-Flight Mass Spectrometry for the Monitoring of Kidney Transplant Recipients**

**Shijian Feng^1^, MD, PhD, Chengfang Xiang^4^,** **MS, Yushi He^1^, MM, Zhuoya Li^2^, MS,** **Zhongjun Zhao****^3^, PhD,** **Bohan Liu^1^, MM,** **Zhaofa Yin^1^, MM,** **Qiyu He^1^, MM,** **Yanting Yang****^3^, BD,** **Zhongli Huang^1^, MD, PhD,** **Tao Lin^1^, MD, PhD, Wenwen Li^2 *^, PhD, Yixiang Duan^3 *^, PhD**

**1. Department of Urology and Institute of Urology (Laboratory of Reconstructive Urology), Organ Transplantation Center, West China Hospital, Sichuan University, Chengdu, People's Republic of China.**

**2.** **West China School of Public Health and West China Fourth Hospital, Sichuan University, Chengdu 610041, People's Republic of China.**

**3. School of Mechanical Engineering,** **Sichuan University, Chengdu 610064, People's Republic of China.**

**4. College of Chemistry,** **Sichuan University, Chengdu 610064, People's Republic of China.**

*** Corresponding author.**

**Wenwen Li:**

**Address:** **No.17 People's South Road, Chengdu, China, 610041.**

**Email:** [**wenwentp@163.com**](mailto:wenwentp@163.com)**;**

**Yixiang Duan:**

**Address: No.24 South Section 1, Yihuan Road, Chengdu, China, 610065.**

**Email:** [**yduan@scu.edu.cn**](mailto:yduan@scu.edu.cn)**;**


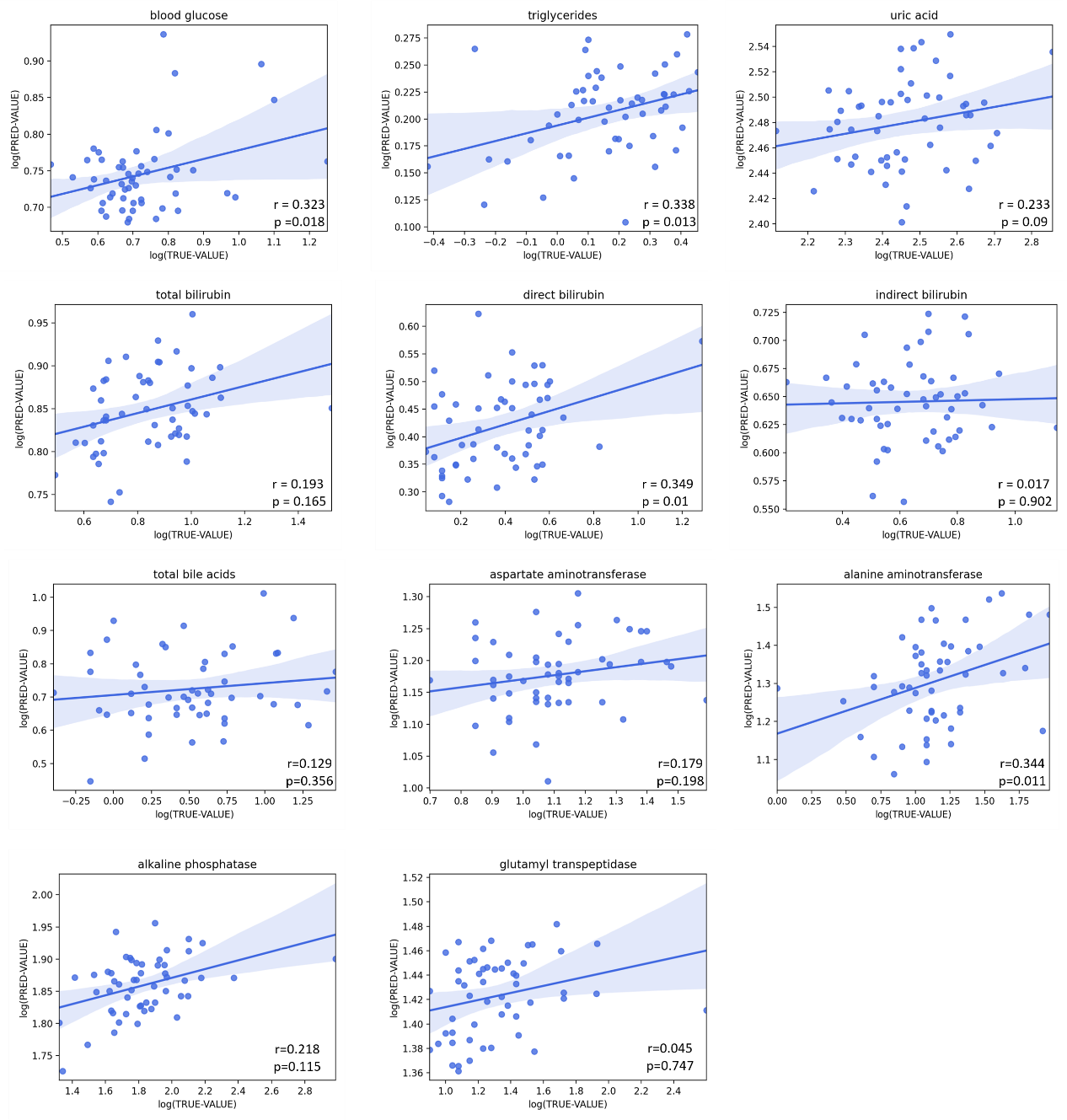


Supplementary Figure 1. Correlations between clinical true values and regression model predicted values of blood glucose, triglycerides, uric acid, total bilirubin, direct bilirubin, indirect bilirubin, total bile, aspartate aminotransferase, alanine aminotransferase, alkaline phosphatase, glutamyl transpeptidase of KTx patients.


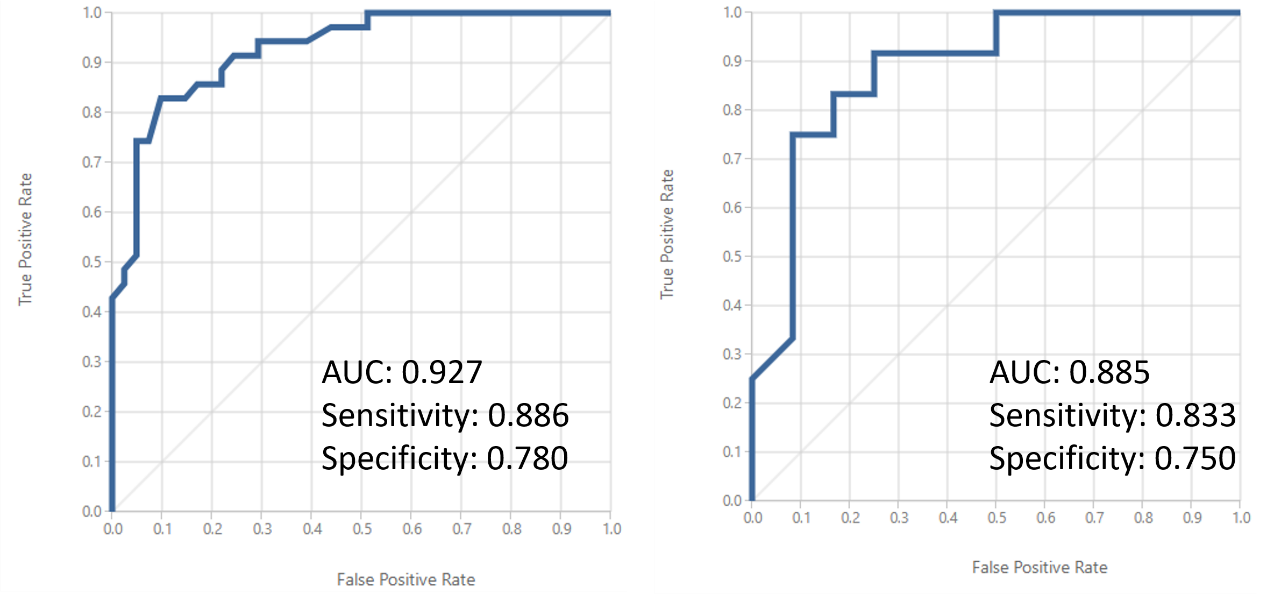


Supplementary Figure 2. RF model for glucose status (135 normal and 40 high) classification by 200% SMOTE resampling (left, 135 normal vs 120 high) and under-sampling (right, 40 normal vs 40 high).


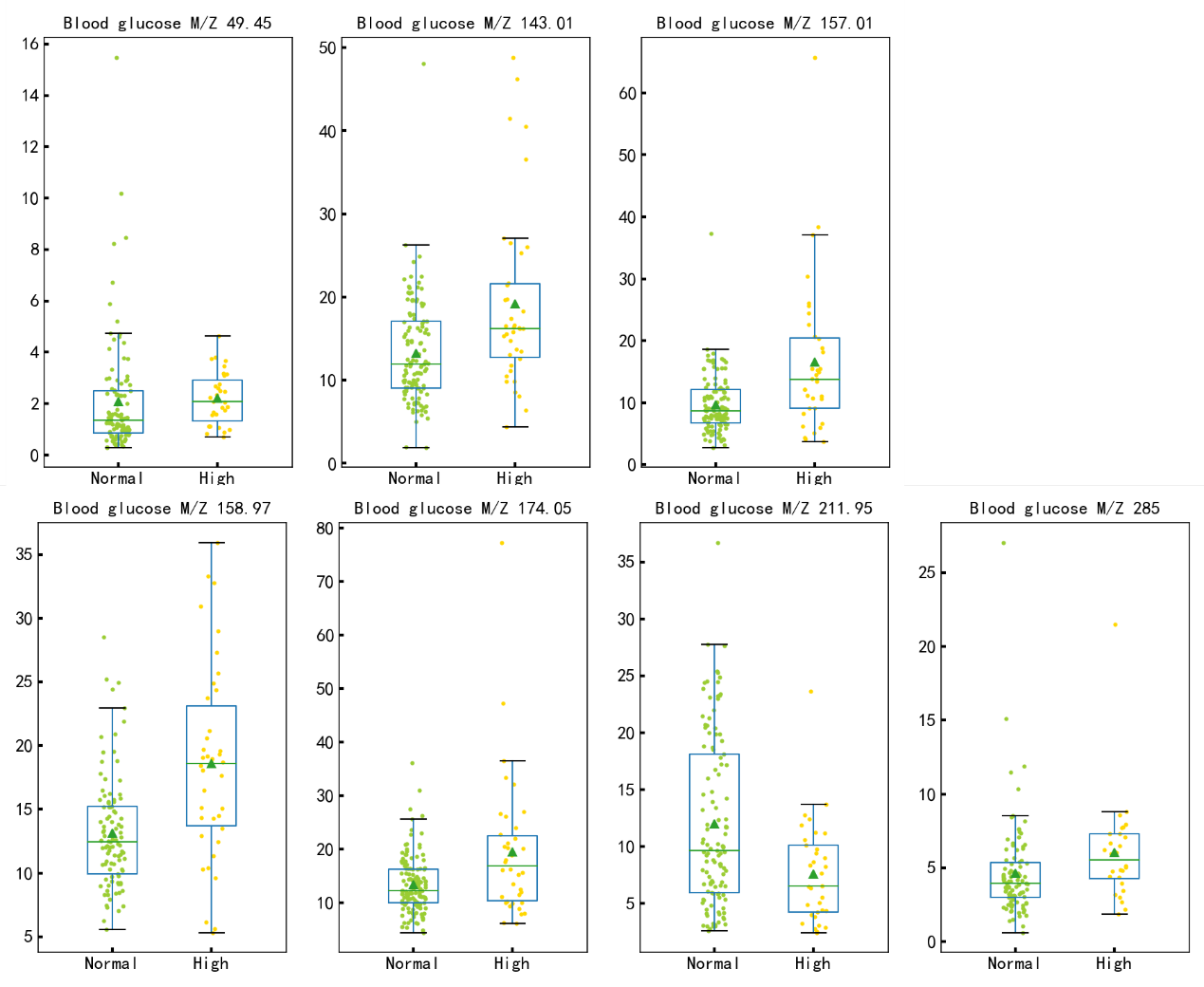


Supplementary Figure 3. Significant exhaled compounds (p<0.01) between normal (<5.9 mmol/L) and high blood glucose status (>5.9 mmol/L).


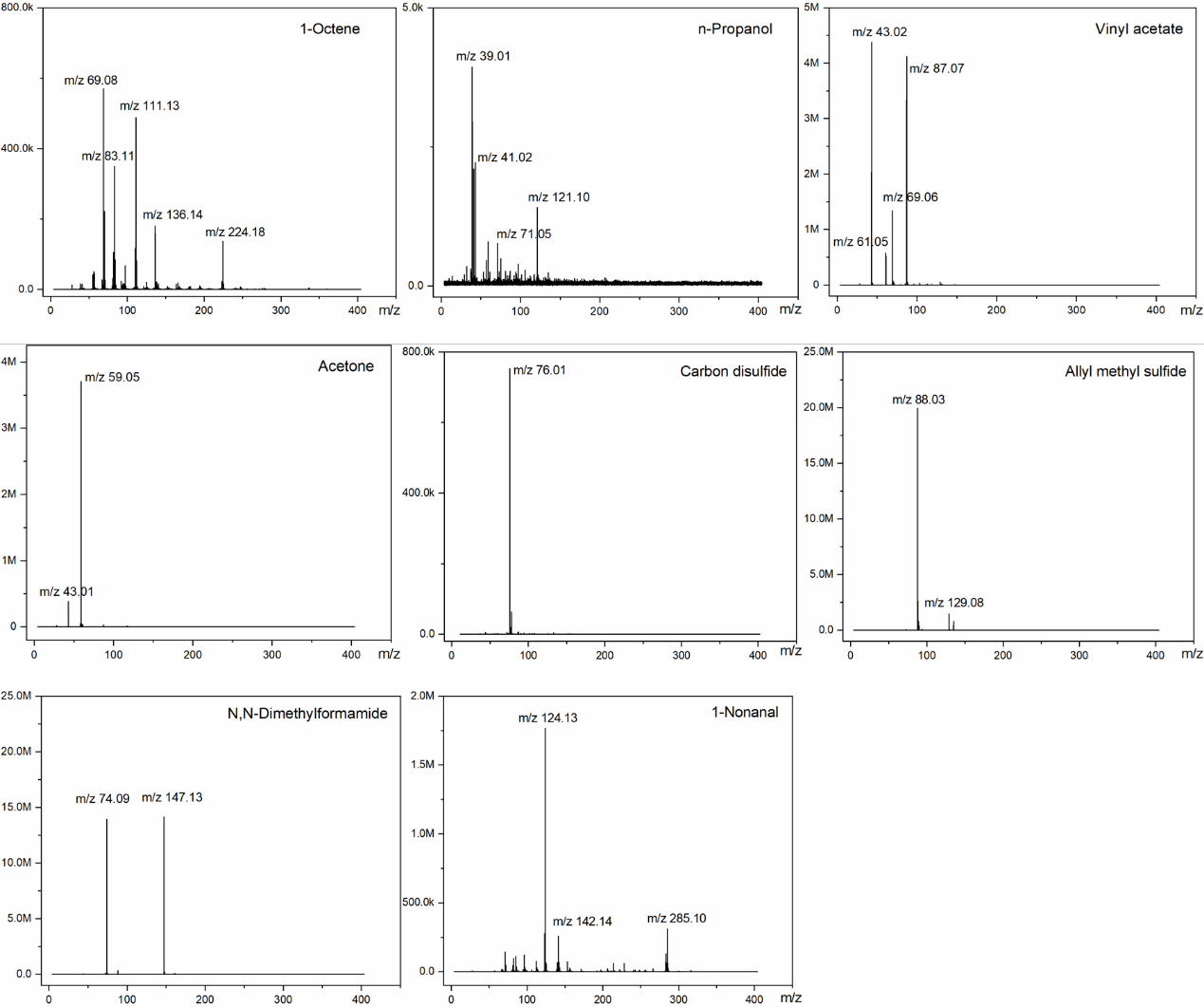


Supplementary Figure 4. Raw mass spectra of standards of significant exhaled compounds.


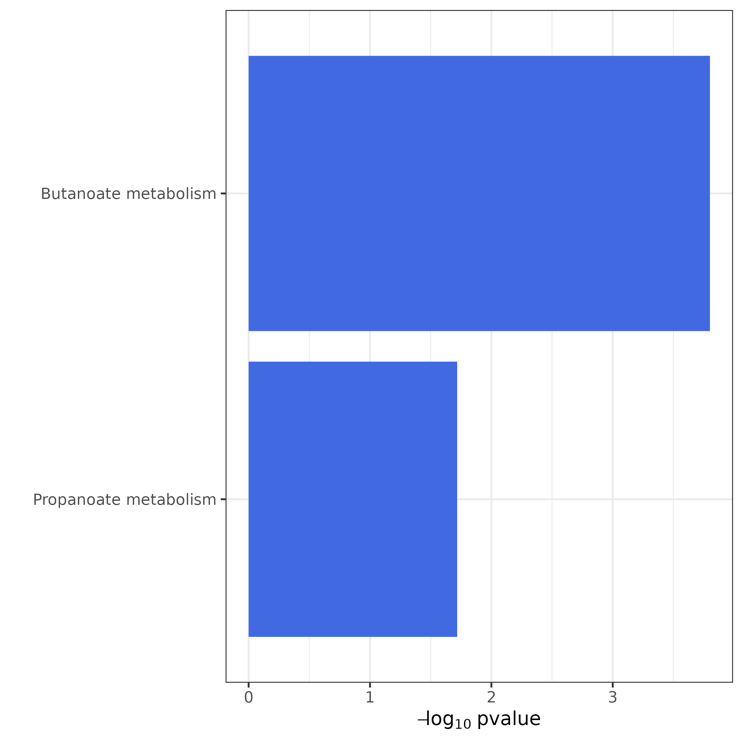


Supplementary Figure 5. KEGG pathway analysis based on different exhaled metabolic contents.


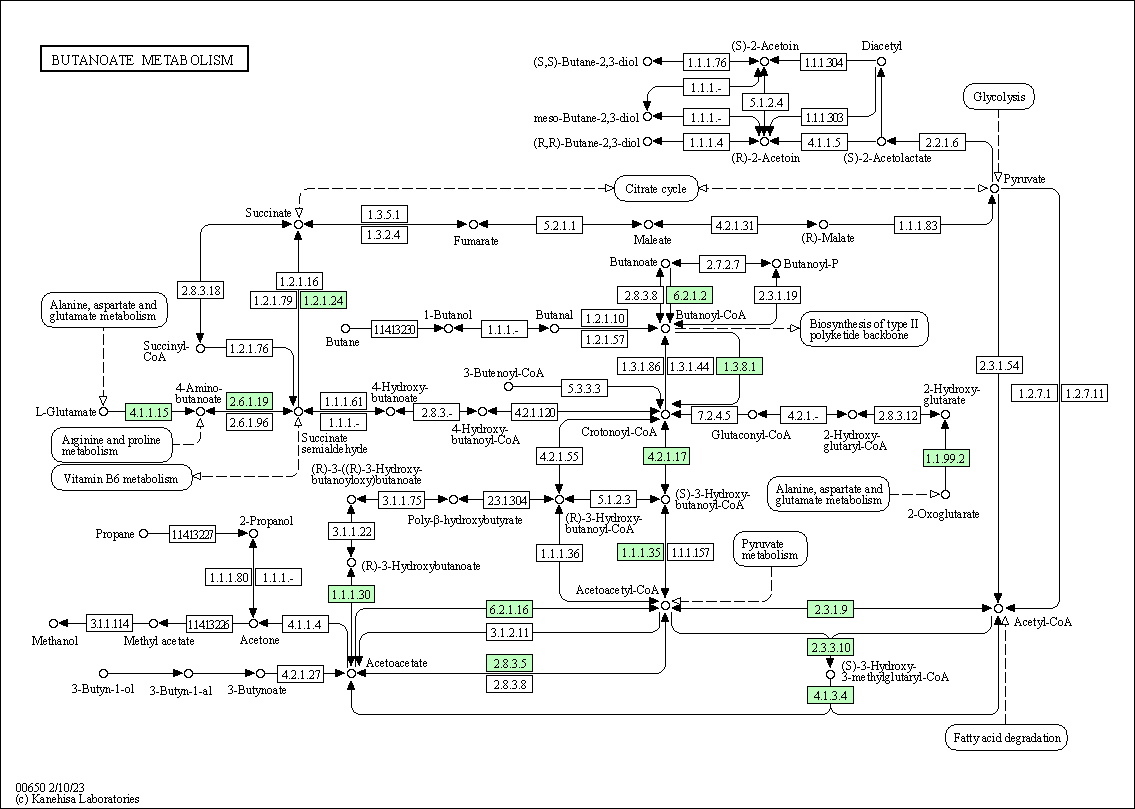
Supplementary Figure 6. Crucial different metabolic contents identified by UVP-TOF-MS involved in butanoate metabolism.


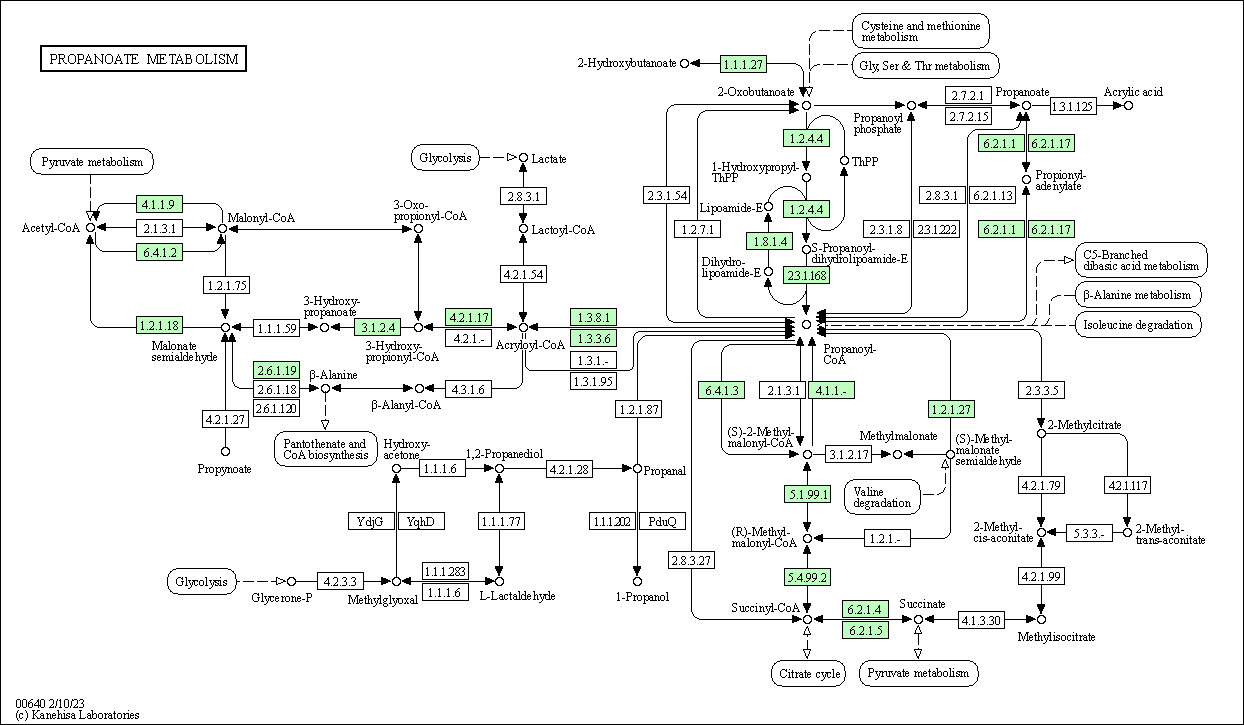


Supplementary Figure 7. Crucial different metabolic contents identified by UVP-TOF-MS involved in propanoate metabolism.
